# Wave reflection and transmission in multiply stented blood vessels

**DOI:** 10.1098/rspa.2017.0015

**Published:** 2017-06-07

**Authors:** T. K. Papathanasiou, A. B. Movchan, D. Bigoni

**Affiliations:** 1 Department of Mechanical, Aerospace and Civil Engineering, Brunel University London, Uxbridge UB8 3PH, UK; 2 Department of Mathematical Sciences, University of Liverpool, Liverpool L69 7ZL, UK; 3 DICAM, University of Trento, Trento 38123, Italy

**Keywords:** elastic waves, fluid–solid interaction, wave reflection, periodic structures, asymptotic analysis

## Abstract

Closed circulatory systems display an exquisite balance between vascular elasticity and viscous fluid effects, to induce pulse-smoothing and avoid resonance during the cardiac cycle. Stents in the arterial tree alter this balance through stiffening and because a periodic structure is introduced, capable of interacting with the fluid in a complex way. While the former feature has been investigated, the latter received no attention so far. But periodic structures are the building blocks of metamaterials, known for their ‘non-natural’ behaviour. Thus, the investigation of a stent's periodic microstructure dynamical interactions is crucial to assess possible pathological responses. A one-dimensional fluid–structure interaction model, simple enough to allow an analytical solution for situations of interest involving one or two interacting stents, is introduced. It is determined: (i) whether or not frequency bands exist in which reflected blood pulses are highly increased and (ii) if these bands are close to the characteristic frequencies of arteries and finally, (iii) if the internal structure of the stent can sensibly affect arterial blood dynamics. It is shown that, while the periodic structure of an isolated stent can induce anomalous reflection only in pathological conditions, the presence of two interacting stents is more critical, and high reflection can occur at frequencies not far from the physiological values.

## 1. Introduction

Vascular elasticity interacts with blood viscosity in the arterial tree during the cardiac cycle to avoid dynamic effects such as resonance, pressure blow-up and collapsing pulses. A stent, implanted for the treatment of vascular stenosis or aneurysm, alters this delicate interaction, so that it affects the local blood flow patterns and constitutes a site of blood pulse-wave reflection [1]. This last effect is due to the increased stiffness and the changes in local blood vessel geometry, characterizing the stented region [2,3]. It depends strongly on the mechanical properties, geometry and the overall design of the stent [4–6]. The compliance characteristics of blood vessel regions, where stents are placed, also differ from those of a healthy tissue due to the presence of atheromatous plaques (stiffening), leading to stenosis (altered geometry characteristics) and finally altered pulse characteristics [7–10].

Typically, stents are much smaller than the principal wavelength of arterial blood pulses and, as a consequence, pulse wave reflection is expected to be small. However, as also mentioned in [1], even the slightest changes in local flow pulsatility may have important physiological consequences. Another important aspect is that stent–pulse wave interactions may alter the blood pulse characteristics even in sites away from the stented region [1,3]. Finally, certain cardiovascular conditions (e.g. atrial fibrillation) are associated with shorter pulse wavelengths [11]. In these cases, increased frequencies manifest, especially when irregularities in the pulse waveform occur and, consequently, higher harmonics are present, so that even the internal structure of the stent might be significant during reflection/transmission. The aim of the present article is to examine the possibility of increased reflection frequency bands due to the placement of a single stent and the interaction of two successive stents, taking into account also the periodicity of the stent structure. Bloch waves in periodic structures play a central role in this endeavour.

The analysis of Bloch waves is common in solid-state physics, problems of photonics and acoustics in periodic multi-scale media [12,13]. In the last decade, the notion of metamaterials has been introduced and developed regarding applications in wave propagation problems (e.g. [14–16]). As illustrated in [14,17], and in the book [18], there exists a formal connection between dispersion properties of waves in infinite periodic media and transmission problems developed for multi-scale structured interfaces [19–21]. Therefore, the presence of a stent in the arterial tree configures in a sense a metamaterial, so that ‘non-natural’ effects typical of these materials might be induced.

In the present paper, an analytical model, which takes into account the fluid–solid interaction in the framework of a transmission problem for a pulsating flow through a stent reinforcing a blood vessel, is proposed. It is known (e.g. [1–3]) that there is a reflection of the wave from the boundary of the stent. However, the connection of the reflection/transmission properties with the microstructure of the periodic stent has not been addressed. Intuitively, it is expected that the stent periodic structure will not affect phenomena in the low-frequency regime due to the large wavelength of the pulses. The important issue of frequency bands affected by the stent periodic structure is also addressed in this paper. Moreover, the interaction between two stents separated by a finite distance is analysed for the first time. It is demonstrated that the reflection and transmission coefficients for a system of stents depend strongly on their distance of separation. Analytical formulae are presented here to evaluate the reflections coefficients, and the transmission resonances are also identified.

A basic feature of this study is the derivation of a simple model, capable of incorporating the effects of the stent microstructure in pulse reflection analysis. Among the desired characteristics of such a model are: low spatial dimensionality, linearity and the minimum number of free parameters. Given these characteristics, several significant blood flow aspects, such as viscosity and local fluid–elastic or rigid body interactions [1,2,22], complicated blood flow patterns and turbulent flow issues [1,2,7], are not addressed. However, the proposed model could provide indications, in terms of critical locations in the arterial tree or critical parameter values, for efficient application of more involved, large-scale, computationally intensive simulations.

The paper is organized as follows: initially, the linear one-dimensional arterial pulse model with variable wave propagation speed is formulated. This model is supplemented by specific forms of pulse wave speed variation within each characteristic cell of the stent (§2). An analytical solution for the time harmonic volumetric flow pulse in a stented region is presented in §3. Subsequently, the reflection–transmission characteristics for a simple stented region are analysed with respect to the pulse frequency and stent properties, including the microstructure (§4). In §5, the case of two successive stents is examined and a parametric analysis with respect to the length of the stented regions and their separation length is conducted. Finally, the findings of the present study are applied to a case of practical interest involving the comparison, in terms of reflection characteristics, of two-stent placement strategies: a single long stent or two successive smaller ones with a narrow gap between them.

## 2. Governing equations

The analysis is based on a simple one-dimensional system simulating pulse transmission through a stented region of a blood vessel. The linearized one-dimensional model derived from averaging and integration, over the blood vessel cross section, of the mass and momentum conservation equations will be adopted [7,23]. The speed of sound will be approximated using essentially the Moens–Korteweg [24,25] model.

### (a) The linearized one-dimensional model

In the case of low Mach number flows and upon neglecting all nonlinear effects (e.g. convective terms, turbulent flow, etc.), several simplified models assume constant pressure *p* and velocity *u* over the blood vessel cross-sectional area *A*. If, in addition, the changes in the cross-sectional area with respect to a reference state are assumed to be very small, i.e. *A*/*A_o_ *≈ 1, the mass and momentum balance equations are [7]

2.1 $$\frac{\partial p}{\partial t}+c^{2}\frac{\rho }{A_{o}}\frac{\partial q}{\partial x}=0$$

and

2.2 $$\frac{\partial q}{\partial t}+\frac{A_{o}}{\rho }\frac{\partial p}{\partial x}=0,$$

where *x* and *t* are the spatial and temporal variable, respectively, *q* = *Au* is the volumetric flow, *c* is the disturbance propagation speed and *ρ* is the fluid density, approximately constant (independent of the pressure) for a nearly incompressible fluid. Assuming sufficient regularity of the volumetric flow and pressure, we can eliminate one of the two fields. Solving for *q*, system (2.1)–(2.2) reduces to the D’ Alembert-type equation

2.3 $$\frac{\partial^{2}q}{\partial t^{2}}-\frac{\partial }{\partial x}\left(c^{2}\frac{\partial q}{\partial x}\right)=0,$$

where, following [3,7], the disturbance propagation speed is approximated as

2.4 $$c^{2}\approx \frac{\beta \sqrt{A_{o}}}{2\rho },$$

with

2.5 $$\beta =\frac{\sqrt{\pi }Eb}{(1-v^{2})A_{o}}.$$

In the definition of *β*, *E and v* are the Young's modulus and Poisson's ratio of the blood vessel tissue, respectively, *b* is the thickness of the blood vessel and *ρ* denotes the density of the blood. Note that in equation (2.3), *c* = *c*(*x*) is assumed to be a function of the spatial variable. This assumption will allow for variable blood vessel properties in the stented area.

Remark Equations (2.1) and (2.2) could also be solved for the pressure *p*, to yield

2.6 $$\frac{\partial^{2}p}{\partial t^{2}}-c^{2}\frac{\partial^{2}p}{\partial x^{2}}=0.$$

In this study, equation (2.3) will be adopted. This form is preferred because the dependent variable appears in divergence form. This form is more suitable for variational methods, such as finite elements. Thus, the analytical solutions that will be derived in this study could constitute a reference for the application and verification of finite-element-based procedures for similar problems.

### (b) A model for the stented region

Inside the stented region, material properties of the blood vessel tissue are bound to differ from those of a healthy part [3,26]. Differences are related not only to the presence of the stent itself but also to the blood vessel condition that dictated the presence of the stent (e.g. arteriosclerosis) [8–10]. With reference to equation (2.4), Young's modulus, blood vessel thickness and diameter are expected to vary. If the speed of sound for the healthy region is denoted by *c*_ref_, the variability in the stented region will be assumed in the form

**Figure 1.**
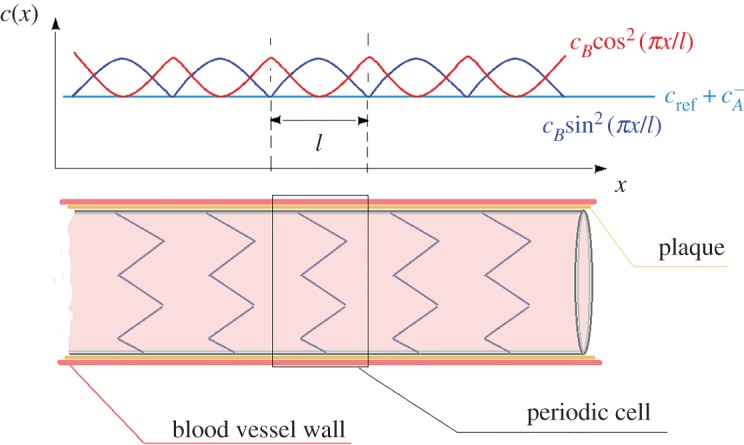
Schematic of the stented area: definition of the periodic cell and assumptions about the disturbance propagation speed variability. (Online version in colour.)

2.7 $$c(x)=c_{\mathrm{ref}}+c_{A}+c_{B}f(x),$$

where *c_A_, c_B_* are constants and *f*(*x*) is a periodic function of period *l*, i.e. *f*(*x* *+* *l*) = *f*(*x*), such that 0 ≤ *f*(*x*) ≤ 1. Constant *c_A_* represents the minimum variation from the healthy region value due to the altered properties of the damaged blood vessel and the stent or stent/graft system (figure 1). Constant *c_B_* is linked to the maximum deviation, measured from the *c*_ref_ *+* *c_A_* state, which occurs within the characteristic cell. This deviation is related to the effect of ‘stiffer’ regions associated with the alloy wire stent grid patterns or the localized presence of alloy wires that constitute the stent grid in a stent/graft system. In the following, assuming that the stent consists of n∈N periodic cells and is confined between *x* = 0 and *x* = *nl*, the specific forms

2.8a $$c(x)=c_{\mathrm{ref}}+c_{A}+c_{B}\sin^{2}\frac{\pi x}{l}$$

and

2.8b $$c(x)=c_{\mathrm{ref}}+c_{A}+c_{B}\cos^{2}\frac{\pi x}{l}$$

will be adopted (figure 1). Using the length *l* of the periodic cell as a characteristic dimension, the non-dimensional variables

2.9 $$\xi =\frac{x}{l}\;\eta =\frac{c_{\mathrm{ref}}t}{l}\;Q=\frac{q}{q_{\mathrm{ref}}},$$

may be introduced. In (2.9), *q*_ref_ represents some reference value of the volumetric flow. Then, equation (2.3) takes the form

2.10 $$\frac{\partial^{2}Q}{\partial \eta^{2}}-\frac{\partial }{\partial \xi }\left({\left(\frac{c}{c_{\mathrm{ref}}}\right)}^{2}\frac{\partial Q}{\partial \xi }\right)=0.$$

Adopting the model presented in the previous section for the stented area, it is

2.11 $$\frac{c(\xi )}{c_{\mathrm{ref}}}=\left\{ \begin{array}{l} 1,\;\xi \in (-\infty ,0)\\ 1+A^{2}+B^{2}\sin^{2}(\pi \xi )\;\mathrm{or}\;1+A^{2}+B^{2}\cos^{2}(\pi \xi ),\;\xi \in (0,n)\\ 1\;,\;\xi \in (n,+\infty ), \end{array}\right.$$

where *A*^2^ = *c_A_*/*c*_ref_ and *B*^2^ = *c_B_*/*c*_ref_. For a typical stent, the definition of the periodic cell and the variability in the disturbance propagation speed are defined schematically in figure 1.

Note further that it is

2.12 $$\int_{0}^{1}\frac{c(\xi )}{c_{\mathrm{ref}}}d\xi =\int_{0}^{1}[1+A^{2}+B^{2}f(\xi )]\;d\xi =1+A^{2}+\frac{B^{2}}{2},$$

for both functions *f*(*ξ*) = sin^2^(*πξ*) and *f*(*ξ*) = cos^2^(*πξ*). Thus, the value

2.13 $$C=1+A^{2}+\frac{B^{2}}{2}$$

is the mean non-dimensional speed in each characteristic cell.

Remark The case *f*(*ξ*) = sin^2^(*πξ*) corresponds to a periodic cell which is more compliant at its edges, whereas the case *f*(*ξ*) = cos^2^(*πξ*) corresponds to a periodic cell which is more compliant at its central region.

## 3. Time-periodic volumetric flow rate in stented regions

In this section, the transmission problem for a blood vessel containing a stented region will be analysed. The closed form solution of the specific problem for the determination of the transmission and reflection coefficients is possible. Assuming solutions of the form *Q* = *y*(*ξ*)e^i*ωη*^, where *ω* is the angular frequency, equation (2.3) becomes

3.1 $$\frac{d}{d\xi }\left({\left[1+A^{2}+B^{2}f(\xi )\right]}^{2}\frac{dy}{d\xi }\right)+\omega^{2}y=0.$$

Differentiating with respect to *ξ* and setting

3.2 $${\left[1+A^{2}+B^{2}f(\xi )\right]}^{2}\frac{dy}{d\xi }=Y,$$

it is

3.3 $$\frac{d^{2}Y}{d\xi^{2}}+{\left[\frac{\omega }{1+A^{2}+B^{2}f(\xi )}\right]}^{2}Y=0.$$

Assuming that *f*(*ξ*) = sin^2^(*πξ*) or *f*(*ξ*) = cos^2^(*πξ*) and applying the formulae 2 sin^2^ *ϑ* = 1−cos2*ϑ* and 2cos^2^ *ϑ* = 1 + cos2*ϑ*, respectively, leads to

3.4 $$\frac{d^{2}Y}{d\xi^{2}}+\frac{\omega^{2}}{C^{2}{\left(1\mp \varepsilon \cos2\pi \xi \right)}^{2}}Y=0,$$

where the minus sign corresponds to the choice *f*(*ξ*) = sin^2^(*πξ*), the plus sign to *f*(*ξ*) = cos^2^(*πξ*) and

3.5 $$\varepsilon =\frac{B^{2}}{2C}=\frac{B^{2}}{2+2A^{2}+B^{2}}<1.$$

Assuming that ε≪1 and since |cos⁡2πξ|≤1, the expansion

3.6 $${\left(1\mp \varepsilon \cos2\pi \xi \right)}^{-2}=1\pm 2\varepsilon \cos2\pi \xi +O(\varepsilon^{2})\approx 1\pm 2\varepsilon \cos2\pi \xi$$

might be adopted. Finally, equation (3.4), using (3.6) and setting $\pi \xi =z\Rightarrow d\xi =\pi^{-1}dz,$ produces the form

3.7 $$\frac{d^{2}Y}{dz^{2}}+\left[{\left(\frac{\omega }{\pi C}\right)}^{2}\pm 2\varepsilon {\left(\frac{\omega }{\pi C}\right)}^{2}\cos2z\right]Y=0.$$

The above equation is the standard Mathieu equation ($Y^{\prime \prime }+[a\pm 2q\cos(2z)]Y=0$), with

3.8 $$\begin{array}{rl} & a={\left(\frac{\omega }{\pi C}\right)}^{2}\\ \mathrm{and}\; & q=\varepsilon {\left(\frac{\omega }{\pi C}\right)}^{2}=\varepsilon a. \end{array}\}$$

The general solution is

3.9 $$Y(\xi )=D_{1}M_{S}\left(a,\varepsilon a,\pi \left(\frac{1}{2}-\xi \right)\right)+D_{2}M_{C}\left(a,\varepsilon a,\pi \left(\frac{1}{2}-\xi \right)\right),$$

for *f*(*ξ*) = sin^2^(*πξ*) and

3.10 $$Y(\xi )=D_{1}M_{S}(a,\varepsilon a,\pi \xi )+D_{2}M_{C}(a,\varepsilon a,\pi \xi ),$$

for *f*(*ξ*) = cos^2^(*πξ*), where M*_S_*, M*_C_* are the Mathieu functions, linearly independent solutions of (3.7). Given *a*, *q* = *ϵa*, there exists a characteristic exponent *µ*, such that (see [27,28])

3.11 $$Y_{\mu }(\xi )=e^{i\mu \xi }g(\xi ),$$

where *g*(*ξ* *+* *π*) = *g*(*ξ*) and *µ* = *µ*(*ϵ,a*) [27].

**Figure 2.**
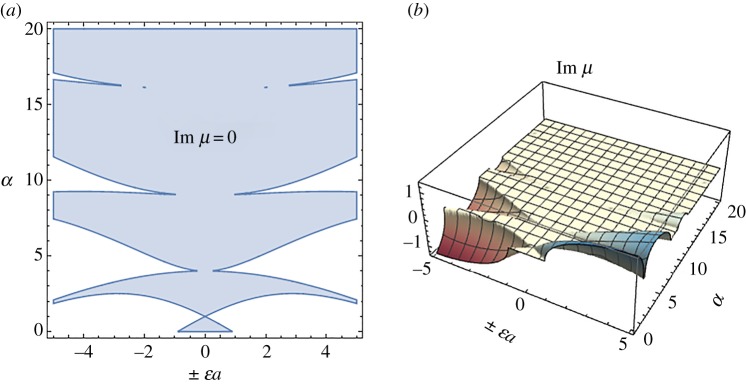
Characteristic exponent region corresponding to periodic solutions (*a*) and characteristic exponent values corresponding to solutions with increasing/decreasing amplitude (*b*). (Online version in colour.)

The case of periodic solutions corresponds to Im *µ* *=* 0 (figure 2*a*). Solutions of increasing amplitude occur if Im *µ* < 0 and solutions of decreasing amplitude if Im *µ > *0 (figure 2*b*). Note that by using both positive and negative values of the term *q* = *ϵa*, in figure 2, both cases *f*(*ξ*) = cos^2^(*πξ*) and *f*(*ξ*) = sin^2^(*πξ*) have been considered.

## 4. The transmission problem for a single-stented region

**Figure 3.**
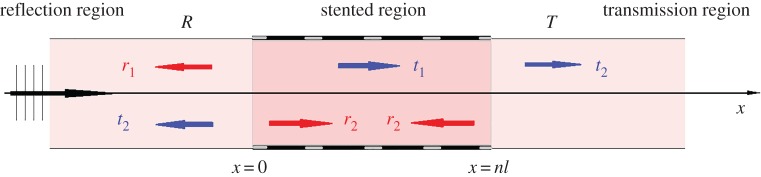
Reflection–transmission phenomenon for the stented area. (Online version in colour.)

In this section, the pulse transmission problem for a blood vessel with a stented area will be analysed. The reflection–transmission scheme in figure 3 is representative of the phenomenon of pulse propagation through a stented area. An incoming pulse moving from the negative *x*-axis towards positive *x*-values reaches the stented area at *x* = 0 and is partially reflected. The portion of the pulse that enters the stented area is again partially reflected at *x* = *nl* (the right-hand end of the stent). Another reflection occurs as the back-propagating pulse (the one reflected at *x* = *nl*) reaches *x* = 0.

In the following, the volumetric flow rate in the reflection region, stented region and transmission region are assumed of the form

4.1 $$\begin{array}{rl} Q_{R} & =q_{R}(\xi )e^{i\omega \eta },\;-\infty <\xi <0. \end{array}$$

4.2 $$\begin{array}{rl} Q & =y(\xi )e^{i\omega \eta },\;0<\xi <n, \end{array}$$

4.3 $$\begin{array}{rl} Q_{T} & =q_{T}(\xi )e^{i\omega \eta },\;n<\xi <\infty , \end{array}$$

respectively, where *ω* is the angular frequency. The flow rate in the reflection region consists of the original incoming wave, with unit amplitude and the reflected wave with amplitude *R*

4.4 $$q_{R}=e^{i\omega \xi }+Re^{-i\omega \xi }=(1+R)\cos\omega \xi +i(1-R)\sin\omega \xi .$$

In the transmission region, the outgoing wave (moving towards *x* =  + ∞) is of amplitude *T*, i.e.

4.5 $$q_{T}=Te^{i\omega \xi }=T\cos\omega \xi +iT\sin\omega \xi .$$

Finally, in the stented region, evoking equation (3.1), we obtain

4.6 $$y=-\frac{1}{\omega^{2}}\frac{dY}{d\xi },$$

where *Y* is one of (3.10) or (3.11), depending on the particular form of *f*(*x*), as prescribed in the previous section. Four constants, namely *R*, *T*, *D*_1_, *D*_2_ appear in total in (4.1), (4.2) and (4.3), taking into account equation (3.10) or (3.11). For the solution of the transmission problem, consisting of the determination of the reflection coefficient |R| and the transmission coefficient |T|, four matching conditions for the fields prescribed at (3.11)–(4.3) are needed. These conditions are obtained by the (i) continuity of the volumetric flow rate *q* and (ii) the continuity of $c^{2}\partial q/\partial x$ appearing in divergence form in equation (2.3), at the interfaces *x* = 0 and *x* = *nl*. The first of these conditions expresses continuity of mass and the second conservation of energy at the interfaces. The four interface conditions (in non-dimensional variables) read

4.7 $$\begin{array}{rl} \frac{dq_{R}}{d\xi } & =Y(\xi )\;\mathrm{and}\;q_{R}(\xi )=-\frac{1}{\omega^{2}}\frac{dY}{d\xi },\mathrm{at}\;\xi =0 \end{array}$$

4.8 $$\begin{array}{rl} \frac{dq_{T}}{d\xi } & =Y(\xi )\;\mathrm{and}\;q_{T}(\xi )=-\frac{1}{\omega^{2}}\frac{dY}{d\xi },\mathrm{at}\;\xi =n. \end{array}$$

Using (4.1)–(4.3), (4.7) and (4.8), a linear system (for the determination of *R*, *T*, *D*_1_, *D*_2_) of the form **Au **= **b** is formulated, where

4.9 $$\text{A}=\left[\begin{array}{cccc} i\omega & 0 & 0 & 1\\ 1 & 0 & \omega^{-2}\pi & 0\\ 0 & i\omega e^{i\omega n} & -M_{S}(n) & -M_{C}(n)\\ 0 & e^{i\omega n} & \omega^{-2}\pi {M^{\prime }}_{S}(n) & \omega^{-2}\pi {M^{\prime }}_{C}(n) \end{array}\right],\;\text{u}=\left[\begin{array}{c} R\\ T\\ D_{1}\\ D_{2} \end{array}\right]\;\mathrm{and}\;\text{b}=\left[\begin{array}{c} i\omega \\ -1\\ 0\\ 0 \end{array}\right],$$

and the ‘canonical’ form of the Mathieu functions has been employed, i.e. $M_{C}(0)=M_{S}^{\prime }(0)=1.$ For the solution of the above system, Cramer's rule may be employed.

### (a) Numerical evaluation of transmission and reflection

Transmission properties of stented areas will be studied with respect to parameters *C*, *ϵ*, characterizing the stiffness magnitude of the stented region, compared to that of a healthy blood vessel and the number of periodic cells n∈N in the stent. Both cases of periodic functions, namely *f*(*x*) = cos^2^(*πx*/*l*) and *f*(*x*) = sin^2^(*πx*/*l*), will be examined. The purpose of this analysis is (i) to determine whether or not there exist frequency bands in which reflection is maximized and (ii) to investigate if these frequency bands are close to the characteristic frequencies of arterial blood pulses. In the case where these frequency bands exist and include characteristic frequencies of arterial blood pulses, the inherent periodic microstructure of a stent might contribute to pulse blockage and reversal. When frequencies of maximal reflection are far from the characteristic pulse frequencies, the periodic structure of the stent is not affecting blood pulse transmission. Physical intuition suggests that the microstructure of the stent will not affect long pulse waves like those appearing in the arterial tree under normal healthy conditions.

**Figure 4.**
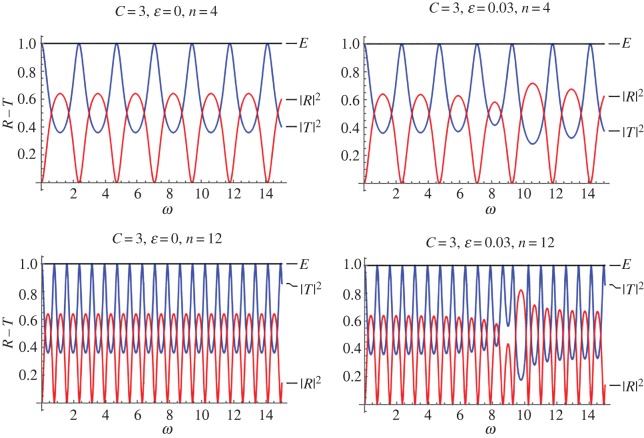
Reflection–transmission diagrams for the single-stent case and different numbers of characteristic cells, in the cases where the internal structure is taken into account; (*ϵ* = 0.03) and not (*ϵ* = 0). In all cases, it is *f* = cos^2^(*πξ*) and *C* = 3. (Online version in colour.)

**Figure 5.**
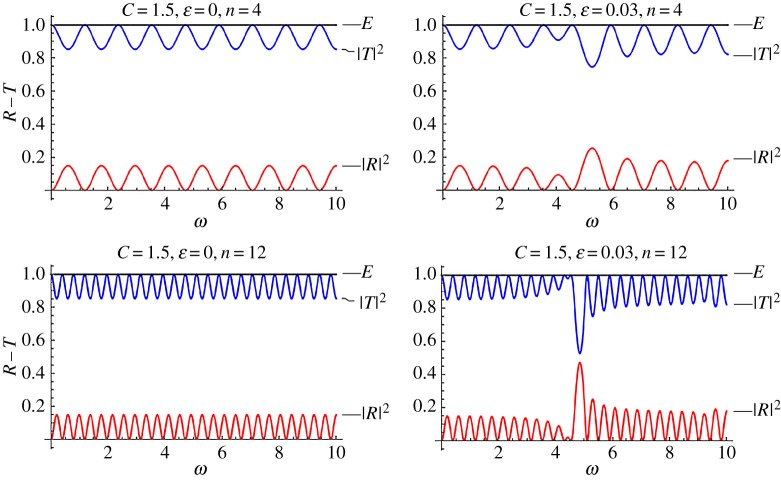
Reflection–transmission diagrams for the single-stent case and different numbers of characteristic cells, in the cases where the internal structure is taken into account; (*ϵ* = 0.03) and not (*ϵ* = 0). In all cases, it is *f* = cos^2^(*πξ*) and *C* = 1.5. (Online version in colour.)

Figures 4 and 5 are reflection–transmission diagrams for a single stented area. In both figures, the case *f* = cos^2^(*πξ*) is considered. The first column, corresponding to *ϵ* = 0, is the case where constant wave speed is assumed along the stent (no internal structure). Figure 4 corresponds to the mean speed *C* = 3, whereas figure 5 corresponds to *C* = 1.5, i.e. a more compliant stent. The squares of the reflection and transmission coefficients are plotted along with the calculated quantity

4.10 $$E=|R{|}^{2}+|T{|}^{2}=1.$$

The above relation expresses the energy conservation in the system considered and provides a further test of the accuracy regarding the computations. Two numbers of characteristic cells inside the stented area have been considered, namely *n* = 4, 12. It can be seen that, in all cases, there is a frequency band where reflection is maximized, for *ϵ* = 0.03 (right column). As the number of the characteristic cells increases (total length of the stent increases), so does reflection. However, the high reflection zone becomes narrower. At the same time, a band of increased transmission occurs before the intense reflection tongue. In all cases, for small values of the non-dimensional frequency *ω*, and in particular for *ω *< 1, no visible changes due to the effect of the stent microstructure occur at the reflection and transmission coefficients.

### (b) Transmission for increased compliance of the stent

Figure 5 corresponds to exactly the same parameters as figure 4, with only *C* being different and equal to 1.5. It is evident, from the second column plots in figure 5, that the maximized reflection band is now displaced towards lower frequencies. This indicates that a very compliant stent, having at the same time an intense internal structure, can be problematic. That is because intense reflection zones tend to appear in lower frequencies. In all cases, frequency bands where transmission characteristics are enhanced appear before the intense reflection tongues.

As increased mean compliance leads to a translation of the intense reflection zones towards lower non-dimensional frequencies, and thus the frequency bands of arterial blood pulses in normal conditions, the limiting case should be examined. Combining equations (2.13) and (3.5), results to

4.11 $$C=\frac{1+A^{2}}{1-\varepsilon }.$$

The limiting case is thus *A* = 0 and *C* = (1 − *ϵ*)^− 1^.

**Figure 6.**
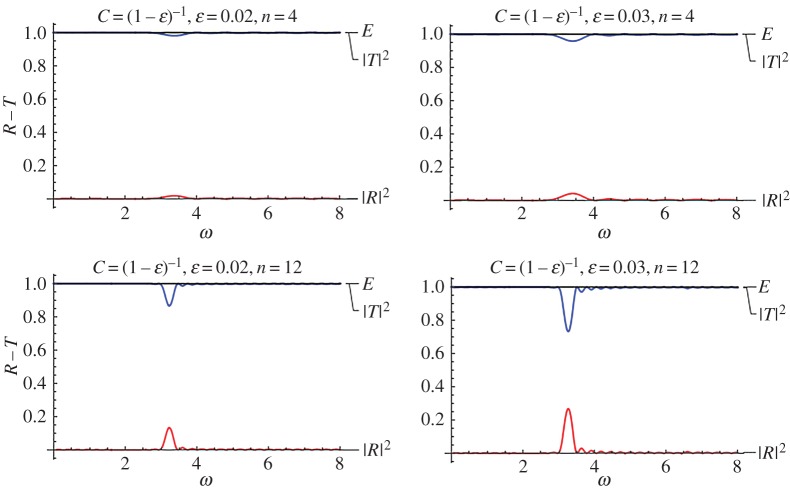
Reflection–transmission diagrams for the single-stent case and different numbers of characteristic cells, for two different values of the internal structure parameter *ϵ* = 0.01, 0.1 in the limiting case of maximized mean compliance *C* = (1 − *ϵ*)^−1^. (Online version in colour.)

Figure 6 is a plot of this scenario for a very small value of the stent internal structure parameter (*ϵ* = 0.02, figure 6*a*) and for a relatively higher value, i.e. *ϵ* = 0.03 (figure 6*b*). The value *ϵ* = 0 is not depicted in this case, as it corresponds to full transmission, given that no stent would actually exist, or it would be infinitely compliant. This situation approximately appears for *ϵ* = 0.02 and *n* = 4, i.e. a small stent with negligible variation in the properties due to its internal structure. As the number of characteristic cells increases, a ‘bump’ in the response appears approximately at *ω* ≈ 3.2.

The reflection–transmission diagram changes significantly as the microstructural parameter *ϵ* increases. Again, as the number of characteristic cells (i.e. the total length of the stent) increases, the high reflection zones become narrower and more intense. However, even in this extreme case, the critical non-dimensional frequency values are very high to affect normal blood pulse waves, which are characterized by much lower frequencies. The intense reflection zones might be significant for higher harmonics of pulse waves related to pathological situations such as atrial flutter or atrial fibrillation.

**Figure 7.**
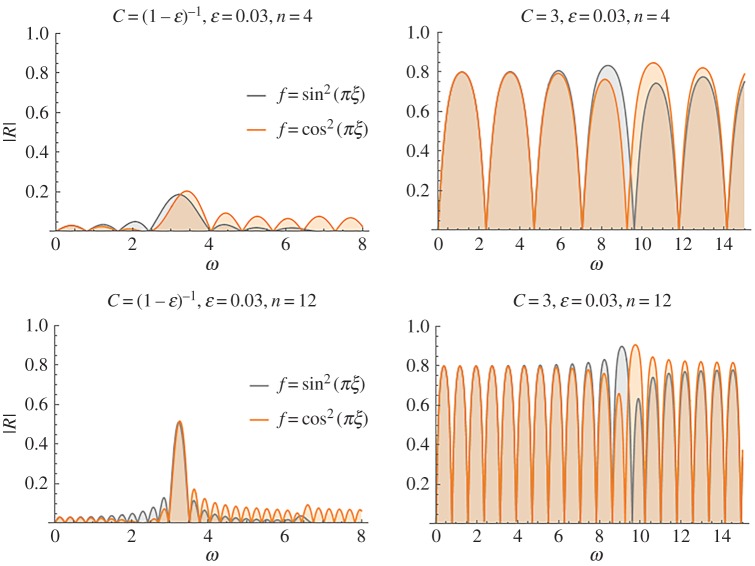
Reflection coefficient for the characteristic cell forms *f*(*ξ*) = cos^2^(*πξ*) and *f*(*ξ*) = sin^2^(*πξ*). (Online version in colour.)

The effect of compliance variation inside the characteristic cell, as expressed by function *f*(*x*) is examined next. The two prescribed cases *f*(*ξ*) = cos^2^(*πξ*) and *f*(*ξ*) = sin^2^(*πξ*) are compared in figure 7, for *ϵ* = 0.03, *n* = 4, 12 and two values of the mean non-dimensional wave speed *C* = 1.5, 3. It is seen that the case *f*(*ξ*) = cos^2^(*πξ*) yields systematically a slightly higher absolute maximum in the reflection coefficient. This is justified by the fact that, in this case, the jump in the overall stiffness, during the transition from the healthy to the stented region, is higher as *f*(*ξ*) = cos^2^(*πξ*) attains it maxima at the ends of the stent (see also figure 1).

The case *f*(*ξ*) = sin^2^(*πξ*) appears to translate high reflection bands at lower non-dimensional frequencies. For this last case, the zone of increased transmission characteristics appears after the high reflection frequency zone. Before this zone, bands of small increase in the reflection appear. The situation is different for *f*(*ξ*) = cos^2^(*πξ*), where the high reflection band precedes the high reflection tongue and slightly increased reflection bands then follow.

**Figure 8.**
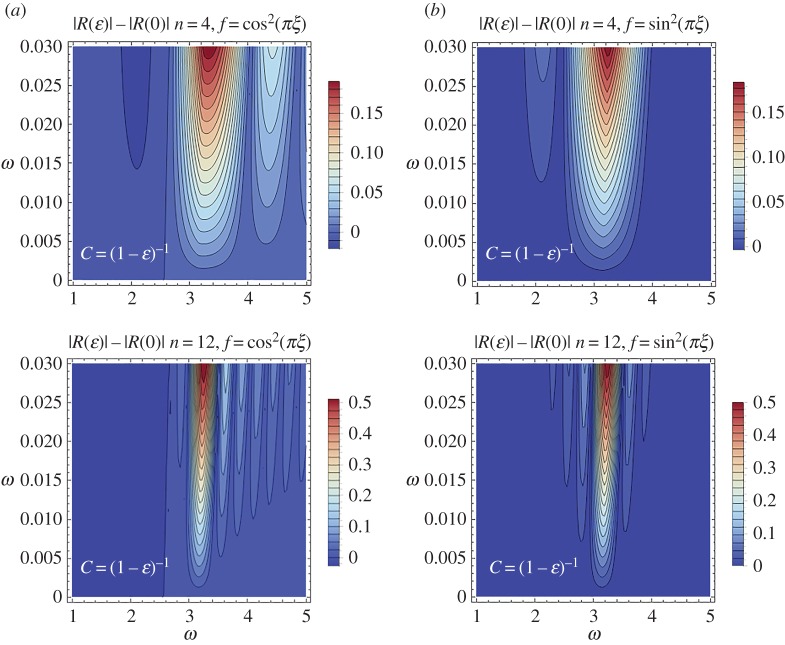
Reflection coefficient difference |R(ε)|−|R(0)| comparison between the stent characteristic cell structure *f*(*ξ*) = cos^2^(*πξ*) (*a*) and *f*(*ξ*) = cos^2^(*πξ*) (*b*). (Online version in colour.)

Finally, contour plots of the effect that the internal structure parameter has on the reflection coefficient are depicted in figure 8, for *f*(*ξ*) = cos^2^(*πξ*) (figure 8*a*) and *f*(*ξ*) = sin^2^(*πξ*) (figure 8*b*). The limit case *C* = (1 − *ϵ*) ^−1^ is examined. The results in figure 8 verify the fact that high reflection zones become narrower and more intense as the number of characteristic cells increase. In addition, it is evident from comparing the first and second column contour plots that increased reflection zones ahead of the main reflection tongue characterize the case *f*(*ξ*) = sin^2^(*πξ*).

The above analysis of the transmission problem for a single stent reveals that the answer to the first point is positive, so that there are frequencies for which reflection becomes very intense. Figures 4–8 indicate that frequency bands exist, within which very high reflection is observed. Regarding the second point, whether these frequency bands are near characteristic frequencies of blood pulses in humans, the following simplified analysis may apply. The non-dimensional cyclic frequency is related to the actual frequency *v_d_* (in Hz), as

4.12 $$\omega =\frac{2\pi lv_{d}}{c_{\mathrm{ref}}}.$$

Thus, the characteristic length of the periodic cell is

4.13 $$l=\frac{\omega c_{\mathrm{ref}}}{2\pi v_{d}}.$$

Based on figure 6, the worst case scenario appears to be *ω* ≈ 3. Typical values for *c*_ref_ are in the order of 4 m s^−1^, so that

4.14 $$l\approx 2v_{d}^{-1}m.$$

It can be concluded from equation (4.7) that the blood pulse frequency must be on the order of hundreds of Hz, when *l* ≈ 4–5 mm. The fact that the typical frequency of blood pulses is approximately 1.3 Hz suggests that the periodicity in the stent structure is not associated with minimization of transmission through a single stented area, at least not in extreme pathological cases. This result is in agreement with the physics of the related phenomena as the blood pulse waves are much longer than typical stent lengths [1,7].

The intense reflection zones, however, could affect higher harmonics in pathological situations (e.g. supraventricular tachycardia [11]), where severe irregularities in the pulse waveforms are present and the energy spectrum spreads to include higher frequencies. Pathological conditions such as atrial flutter or atrial fibrillation are characterized by pulses of frequency much larger than the normal (for supraventricular tachycardia 250 or more pulses per minute can occur). In addition, the associated pulse waveform is highly irregular, suggesting the presence of higher harmonics. In these cases, more sophisticated models, able to represent accurately short pulse waves and perhaps nonlinear effects, should be applied. However, the proposed model is a fast and reliable approach to provide guidelines for efficient application of large-scale, computationally intensive simulations.

## 5. The transmission problem for two successive stented regions

**Figure 9.**
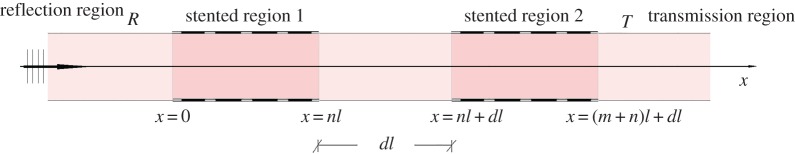
Reflection–transmission phenomenon for two successive stented regions. (Online version in colour.)

In this section, the pulse transmission problem for a blood vessel with two successive stented areas will be analysed. The reflection–transmission scheme in figure 9 is representative of the pulse propagation phenomenon through a series of stented regions. An incoming pulse moving from the negative *x*-axis towards positive *x*-values reaches the first stented region at *x* = 0 and is partially reflected. The portion of the pulse that enters the stented region is again partially reflected at *x* = *nl* (the right-hand end of the first stent). The pulse, that subsequently propagates inside the region between the two stents, reaches the second stented region, at point *x* = *nl* + *d*, where it is partially transmitted and reflected. The reflection wave returns to the first stented region and again reflection and transmission occurs. Finally, the wave that travels inside the second region undergoes another reflection as it exits, at *x* = (*n* + *m*)*l* + *d* and progresses to the transmission region. The overall configuration, consisting of several partially reflecting interfaces, is expected to produce a ‘richer’ reflection coefficient diagram than that representing the case of a single stent. That is, bands of increased reflection are expected to appear in lower frequencies, thus possibly affecting lower harmonics of the arterial pulse spectrum.

In the following, the volumetric flow rate in the reflection region, stented regions, intermediate region and transmission region are assumed of the form

5.1 $$\begin{array}{rl} Q_{R} & =q_{R}(\xi )e^{i\omega \eta },\;-\infty <\xi <0, \end{array}$$

5.2 $$\begin{array}{rl} Q_{1} & =y_{1}(\xi )e^{i\omega \eta },\;0<\xi <n, \end{array}$$

5.3 $$\begin{array}{rl} Q & =v(\xi )e^{i\omega \eta },\;n<\xi <n+d, \end{array}$$

5.4 $$\begin{array}{rl} Q_{2} & =y_{2}(\xi )e^{i\omega \eta },\;n+d<\xi <n+m+d \end{array}$$

5.5 $$\begin{array}{rl} \mathrm{and}\;Q_{T} & =q_{T}(\xi )e^{i\omega \eta },\;n<\xi <\infty , \end{array}$$

respectively. The flow rate wave in the reflection and transmission regions are the same as those appearing in the case of a single stent. In the intermediate region, it is

5.6 $$v(\xi )=D_{3}e^{-i\omega \xi }+D_{4}e^{i\omega \xi }.$$

In the stented regions, the same approximation as that used for a single stent is employed. The constants for the first stented regions are *D*_1_, *D*_2_, while the two constants for the second stented region are *D*_5_, *D*_6_.

Eight constants will appear in total in the analysis for the two stented regions. For the solution of the transmission problem, and the determination of the reflection coefficient |R| and transmission coefficient |T|, eight matching conditions are needed. These conditions are obtained again by the continuity of the volumetric flow rate and the continuity of $c^{2}\partial q/\partial x,$ appearing in divergence form in equation (2.3), at the interfaces *x* = 0 and *x* = *nl*, *x* = (*n* + *d*)*l* and *x* = (*n* + *m* + *d*)*l*. The eight interface conditions (in non-dimensional variables) read

5.7 $$\begin{array}{rl} \frac{dq_{R}}{d\xi } & =Y_{1}(\xi )\;\mathrm{and}\;q_{R}(\xi )=-\frac{1}{\omega^{2}}\frac{dY_{1}}{d\xi },\mathrm{at}\;\xi =0, \end{array}$$

5.8 $$\begin{array}{rl} \frac{dv}{d\xi } & =Y_{1}(\xi )\;\mathrm{and}\;v(\xi )=-\frac{1}{\omega^{2}}\frac{dY_{1}}{d\xi },\;\mathrm{at}\;\xi =n \end{array}$$

5.9 $$\begin{array}{rl} \frac{dv}{d\xi } & =Y_{2}(\xi )\;\mathrm{and}\;v(\xi )=-\frac{1}{\omega^{2}}\frac{dY_{2}}{d\xi },\;\mathrm{at}\;\xi =n+d \end{array}$$

5.10 $$\begin{array}{rl} \mathrm{and}\;\frac{dq_{T}}{d\xi } & =Y_{2}(\xi )\;\mathrm{and}\;q_{T}(\xi )=-\frac{1}{\omega^{2}}\frac{dY_{2}}{d\xi },\;\mathrm{at}\;\xi =m+n+d. \end{array}$$

Using these equations, a linear system (for the calculation *D*_*i*_, *i* = 1, 2, …, 6, *R* and *T*) of the form **Au** = **b** is formulated. The explicit form of the system can be found in appendix A.

### (a) Transmittance of the two-stent system

A parametric study regarding the reflection and transmission characteristics of a two-stent system will be conducted. In the following analysis, the case *f*(*x*) = cos^2^(*πξ*) will be considered. For the two-stent system response, several control parameters exist. Apart from the stent compliance moduli *C* and *ϵ*, there are the number of periodic cells in the first stent n∈N, the number of periodic cells in the second stent m∈N and the distance between the two stented regions *d*.

**Figure 10.**
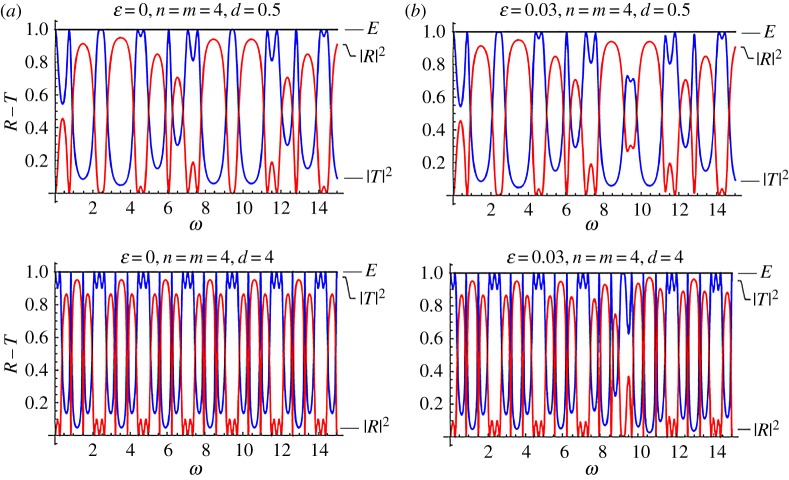
Reflection–transmission diagrams for two stents with *n* = *m* = 4, and different values of *d*, *ϵ*. In all cases, it is *f* = cos^2^(*πξ*), *C* = 3. (Online version in colour.)

**Figure 11.**
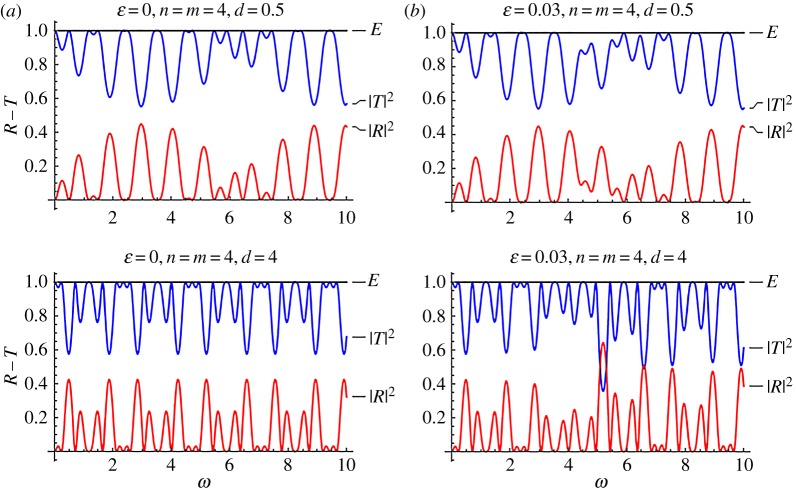
Reflection–transmission diagrams for two stents with *n* = *m* = 4, and different values of *d*, *ϵ*. In all cases, it is *f* = cos^2^(*πξ*), *C* = 1.5. (Online version in colour.)

Figures 10 and 11 are reflection transmission diagrams for the cases of the mean non-dimensional wave speed *C* = 3 and *C* = 1.5. In both cases, the number of periodic cells for each stent is *n* = *m* = 4. The case where no effects of the microstructure are considered, that is *ϵ* = 0, is presented in column *a*. The respective R–T diagrams for *ϵ* = 0.03 are depicted in column *b*. The parameter varying at each row is the distance between the two stents *d*. The plots at the first and second row in figures 10 and 11 correspond to the choices *d* = 0.5, 4, respectively.

Figure 10 is suggestive of the implications induced by the presence of two stents successively placed in a blood vessel and having distance *d* between them. Though the effect of increasing *ϵ* is very small and again enhances reflection at high frequencies, the effect of altering the distance between the stents is dramatic, even in the low-frequency regime. Bands of maximized reflection, with values even higher than |R|=0.9 appear in the non-dimensional frequency range 0 < *ω* < 1. As the distance between the two stents increases, the first reflection coefficient peak decreases, while the following high reflection zone moves to lower frequencies. The presence of variability in the stent properties influences the response in very high non-dimensional frequencies (*ω* ≈ 10).

### (b) Influence of the stent compliance on the transmittance of the two-stent system

The influence of the stent microstructure is more evident in the case *C* = 1.5, shown in figure 11, referring to a two-stent geometry. In this case, the stents are more compliant, compared to that characterized by *C* = 3, and the effect of increased *ϵ* becomes more significant. For *d* = 4, an increased reflection zone appears approximately at *ω* ≈ 5.

**Figure 12.**
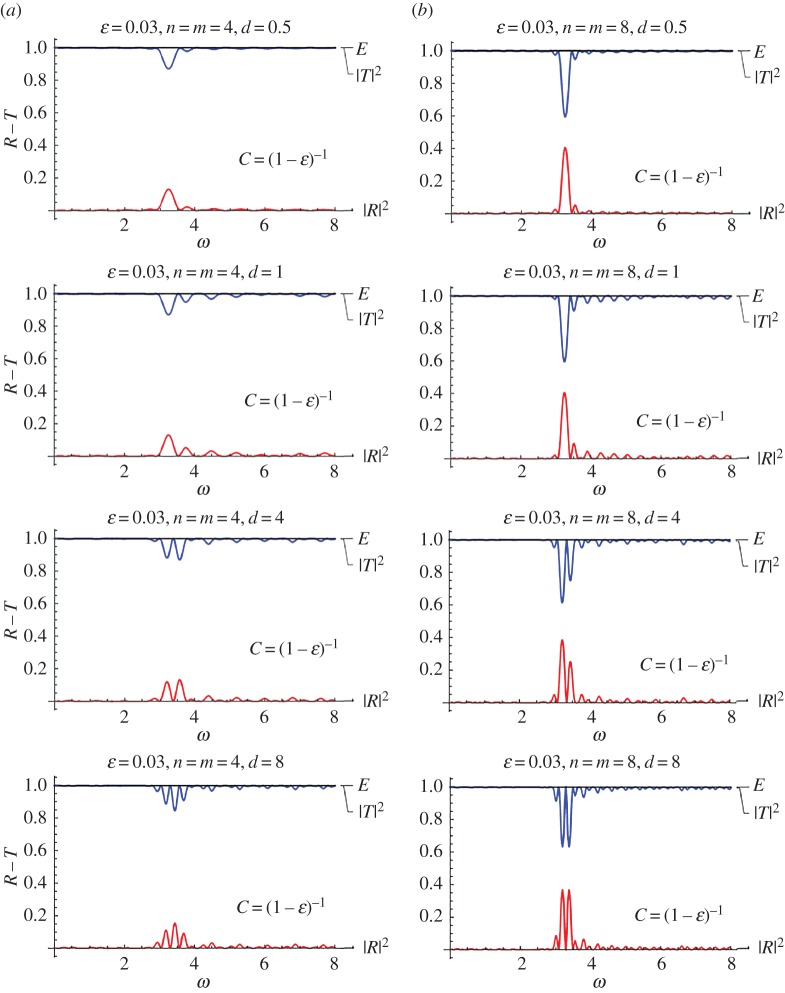
Reflection–transmission diagrams for two stents with *n* = *m* = 4, and different values of *d*. It is *f* = cos^2^(*πξ*), *C* = (1 − *ϵ*)^−1^ and *ϵ* = 0.03. (Online version in colour.)

The limit case *C* = (1 − *ϵ*)^−1^ of a very compliant stent, where the influence of the periodic structure is crucial, is analysed in the following. Results for this case are shown in figure 12. The values *d* = 0.5, 1, 4 and 8 are considered. In all cases, it is *ϵ* = 0.03. Finally, the number of periodic cells in the stents is *n* = *m* = 4 for figure 12*a*, while it is *n* = *m* = 8 for figure 12*b*. In all cases ‘bumps’ in the reflection–transmission coefficient curves appear around the non-dimensional frequency *ω* ≈ 3. The amplitude of these spikes increases with increase in the number of periodic cells. As the distance *d* between the two stents increases, more spikes of lower amplitude appear. The main spikes at the same time become narrower.

**Figure 13.**
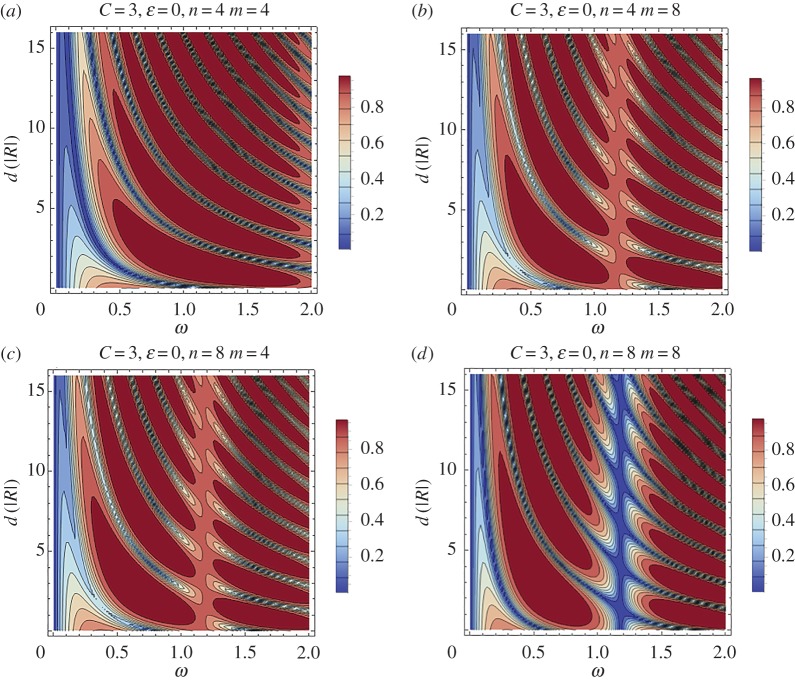
Contour plot of the reflection coefficient for the case of two successive stents as a function of the frequency *ω* and the non-dimensional distance between them *d*. The stent has *C* = 3 and no periodic structure (*ϵ* = 0). (Online version in colour.)

**Figure 14.**
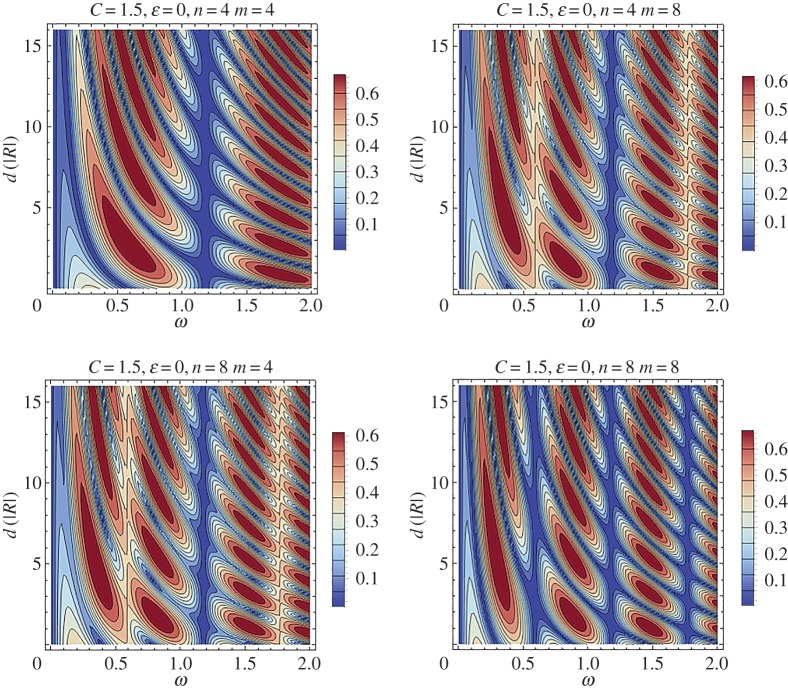
Contour plot of the reflection coefficient for the case of two successive stents as a function of the frequency *ω* and the non-dimensional distance between them *d*. The stent has *C* = 1.5 and no periodic structure (*ϵ* = 0). (Online version in colour.)

The significant effect of the distance *d* between the two stents will be studied in more detail. For this purpose the case of no periodic structure inside the stents is assumed and thus *ϵ* is set to zero. Contour plots of the reflection coefficient as a function of the non-dimensional frequency *ω* and the distance between the stents *d* are depicted in figures 13 and 14. Figure 13 corresponds to a relatively ‘stiff’ stent with *C* = 3.

Figure 14 contains similar results for the case of a more compliant stent with *C* = 1.5. In both cases four different combinations of the stent lengths are examined. These combinations are: (i) *n* = *m* = 4, (ii) *n* = 4 and *m* = 8, (iii) *n* = 8 and *m* = 4 and (iv) *n* = *m* = 8. In all cases, the regime of relatively low non-dimensional frequencies is studied. Figure 13, corresponding to *C* = 3, indicates that a very narrow band exists, characterized by high transmission at very low frequencies, i.e. *ω* < 0.05_,_ and occurring for all values of *d*. Regarding the configuration with *n* = *m* = 4, for relatively higher frequencies, increase in *d* leads to the formation of high reflection zones with intermediate narrow enhanced transmission bands. For a given frequency, as *d* increases, the high reflection zones become narrower. An important observation is that the diagrams for *n* = 4 and *m* = 8 (figure 13*b*) and for *n* = 8 and *m* = 4 (figure 13*c*) are identical. This fact suggests that, at least in this frequency range, the reflection–transmission characteristics of a two-stent system with different stents do not depend on which of the stents appears first. This observation might be important for practical situations when the small or large stents need to be placed at a specific location. As the number of periodic cells increases (case *n* = *m* = 8), a narrow, enhanced transmission band is formed approximately at *ω* = 1.2 for all values of *d*. High reflection branches converge to this zone from lower and higher frequency regions.

Similar patterns characterize the case of a more compliant stent with *C* = 1.5, depicted in figure 14. For the more compliant stent, the enhanced transmission band at *ω *≈ 1.2 appears in all of the cases considered. Additionally, other high reflection zones appear in the case *n* = *m* = 8, at *ω* ≈ 0.6 and *ω* ≈ 1.8. The general rule is that these narrow high reflection bands appear in lower frequencies as the length of the stents and/or the compliance increases. Again, for *C* = 1.5, the reflection–transmission characteristics of the two-stent system with different stents do not depend on which of the stents appears first.

It is important finally to mention the relation between the case of the two stents with the small gap between them and the Fabry–Pérot-type reflector configurations [29,30]. Qualitatively similar phenomena regarding the study of water wave propagation over patches of undulating seabed profiles with a gap between them have been studied recently in [31].

## 6. Discussion and conclusion: a long or two successive smaller stents?

The effect of the stent periodic structure on pulse reflection and transmission coefficients, as well as the interaction of two successively placed stents has been investigated. It has been established that the periodic structure inside a stent can create high reflection phenomena at high frequencies. This could potentially affect higher harmonics of irregular pulses in pathological cases. The case of two successively placed stents is more critical, as high reflection zones appear in relatively low frequencies. The gap between the stents influences significantly the reflectance and the corresponding range of frequencies.

**Figure 15.**
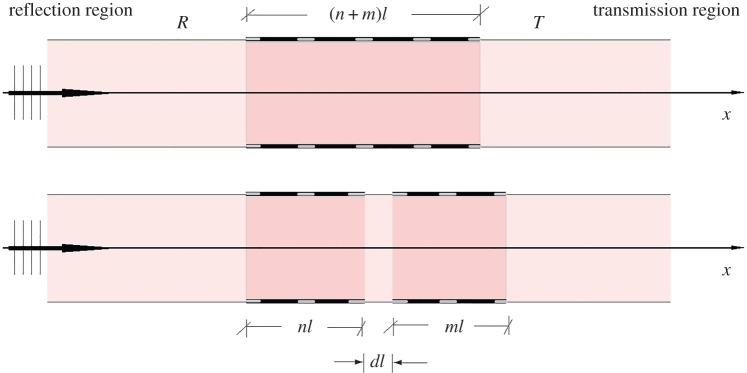
Treatment with a long or two successive small stents featuring a small gap of length *d* between them. (Online version in colour.)

In this section, a test case is presented where the arterial pulse wave reflection is investigated at a blood vessel region, treated with either one long stent or two smaller stents placed one after the other featuring a very small gap between them (see also figure 15). The objective is to study which strategy is preferable in terms of reflection–transmission characteristics. The optimum strategy of course depends on several other important factors, which include alteration of local blood flow patterns, re-blockage effects, as well as the mechanical response of the vessel wall and facilitation of the operation procedure.

The only criterion adopted in the following parametric analysis will be the minimization of the reflection coefficient. The parameters to be considered are: the mean value stent compliance as indicated by *C*, the magnitude of the periodicity in the internal structure *ϵ*, the number of periodic cells in the two successive stents and the gap between them. In all cases, the long stent will consist of 10 periodic cells. The total length of the two successive stents will be exactly the same.

**Figure 16.**
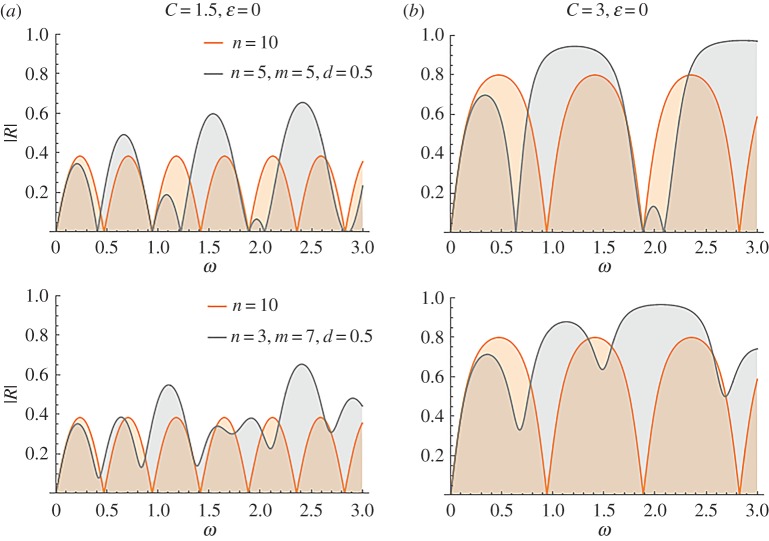
Comparison of reflection coefficients |R| for the cases of a single and two successive stents. Column (*a*) corresponds to more compliant stents. (Online version in colour.)

The effect of the stent compliance and the number of characteristic cells in the two successive stents is analysed in figure 16. The reflection coefficient for *C* = 1.5 and *d* = 0.5 is shown in figure 16*a*. The first row corresponds to the selection *n* = 5, *m* = 5 and the second to the selection *n* = 3, *m* = 7. The analysis of the previous section suggests that the symmetric cases where *n* > *m* yield identical reflection coefficients and therefore need not be examined. Figure 16*b* depicts the case of a less compliant stent (*C* = 3) for the same set of parameters. As expected, the reflection coefficients attains higher maxima for the case *C* = 3.

Figure 16 verifies that the cases of the single long stent and the two successive stents are qualitatively and quantitatively different. The irregularities that appear when two stents are present include both high reflection bands, where the reflection coefficient is almost twice the one corresponding to the single stent case, and enhanced transmission zones.

Two important conclusions are to be drawn. First, at very low frequencies both cases lead to the same reflection coefficient values. Second, the single stent case yields higher values of the reflection coefficient in the vicinity of the lowest frequency reflection tongue. In that sense, the case of the two stents might be favourable. However, the next high reflection tongues are less intense in the case of the single stent. Thus, if higher harmonics are considered, the two-stent case might lead to significant increase regarding the reflection observed.

**Figure 17.**
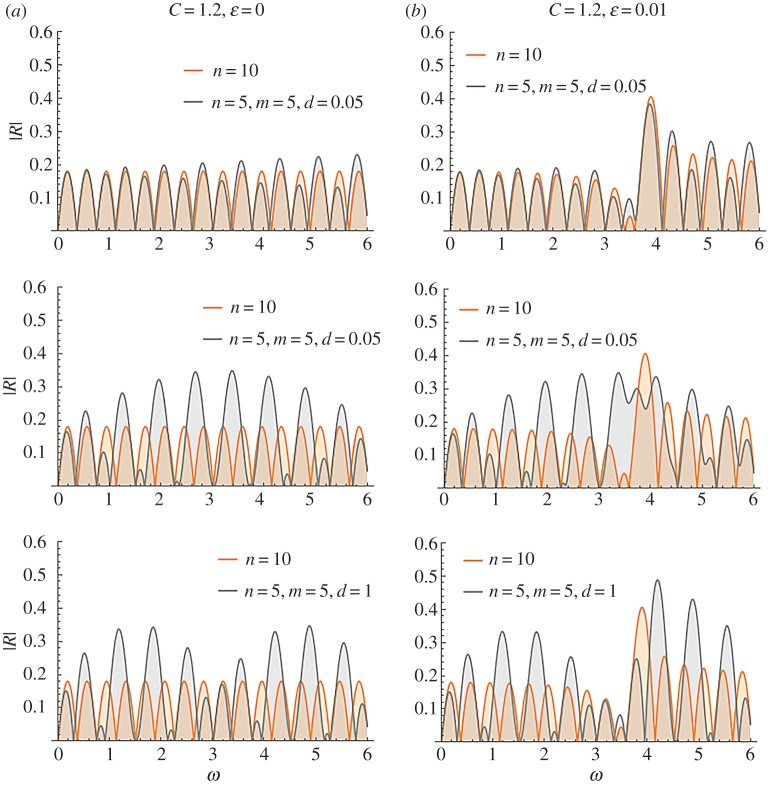
Comparison of reflection coefficients |R| for the cases of a single and two successive stents for the case of a very compliant stent. The effect of the periodic structure of the stents is depicted in (*b*). (Online version in colour.)

The influence of the stent periodic structure is examined in figure 17, for both strategies. A very compliant stent (*C* = 1.2) is considered. Figure 17*a* is the reference case with *ϵ* = 0 and figure 17*b* depicts the case *ϵ* = 0.01, where the periodic microstructure is present. Each row corresponds to a different value of the distance *d. For low non-dimensional frequencies,* i.e. *ω *< 0.5*, the two-stent approach appears to be favourable in terms of reduced reflection*. The first reflection coefficient peak reduces with increase in distance *d*, up to *d* = 1. *However, if higher frequencies are considered* (0.5 < *ω* < 0.8*), the single-stent treatment is better*. This is because increased reflection tongues appear in this frequency range for the two-stent system. Their magnitude increases with increase in *d*. After this frequency range, which is the most critical in terms of applications, the two-stent system produces a succession of high transmittance and high reflection zones. The intensity of these zones increases with increase in *d*. The stent internal structure affects only very high frequencies.

As a conclusion, it can be stated that the use of two successive stents might be favourable in low frequencies, if the gap between them is appropriately selected. However, this approach must be carefully analysed and designed, as a shift to slightly higher frequencies might produce the opposite result. Future studies, using the present model, could be employed for a more detailed analysis of transmittance characteristics in specific frequency bands. These studies will provide more precise indications about case-dependent, optimum treatment strategies.

## Appendix A

The linear system for the determination of the reflection and transmission coefficients in the case of two successive stented regions is **Au** = **b**, with

A 1 $$\begin{array}{r} \text{A}=\left[\begin{array}{cccccccc} \text{i}\omega & 0 & 0 & 1 & 0 & 0 & 0 & 0\\ 1 & 0 & \omega^{-2}\pi & 0 & 0 & 0 & 0 & 0\\ 0 & 0 & M_{S}(n) & M_{C}(n) & -i\omega e^{i\omega n} & i\omega e^{-i\omega n} & 0 & 0\\ 0 & 0 & \omega^{-2}\pi {M^{\prime }}_{S}(n) & \omega^{-2}\pi {M^{\prime }}_{C}(n) & e^{i\omega n} & e^{-i\omega n} & 0 & 0\\ 0 & 0 & 0 & 0 & -i\omega e^{i\omega n_{1}} & i\omega e^{-i\omega n_{1}} & M_{S}(n_{1}) & M_{C}(n_{1})\\ 0 & 0 & 0 & 0 & e^{i\omega n_{1}} & e^{-i\omega n_{1}} & \omega^{-2}\pi {M^{\prime }}_{S}(n_{1}) & \omega^{-2}\pi {M^{\prime }}_{C}(n_{1})\\ 0 & -i\omega e^{-i\omega n_{2}} & 0 & 0 & 0 & 0 & M_{S}(n_{2}) & M_{C}(n_{2})\\ 0 & e^{i\omega n_{2}} & 0 & 0 & 0 & 0 & \omega^{-2}\pi {M^{\prime }}_{S}(n_{2}) & \omega^{-2}\pi {M^{\prime }}_{C}(n_{2}) \end{array}\right], \end{array}$$

A 2 $$\begin{array}{rl} \text{u} & ={\left[\begin{array}{cccccccc} R & T & D_{1} & D_{2} & D_{3} & D_{4} & D_{5} & D_{6} \end{array}\right]}^{T}, \end{array}$$

A 3 $$\begin{array}{rl} \mathrm{and}\;\text{b} & ={\left[\begin{array}{cccccccc} i\omega & -1 & 0 & 0 & 0 & 0 & 0 & 0 \end{array}\right]}^{T}, \end{array}$$

where *n*_1_ = *n* + *d* and *n*_2_ = *n* + *m* + *d,* and the ‘canonical’ form of the Mathieu functions has been employed, i.e. *M_C_*(0) = *M*′*_S_*(0) = 1.
